# Dpep Inhibits Cancer Cell Growth and Survival via Shared and Context-Dependent Transcriptome Perturbations

**DOI:** 10.3390/cancers15225318

**Published:** 2023-11-07

**Authors:** Qing Zhou, Lloyd A. Greene

**Affiliations:** Department of Pathology and Cell Biology, Columbia University Vagelos College of Physicians and Surgeons, New York, NY 10032, USA; qz2266@cumc.columbia.edu

**Keywords:** Dpep, ATF5, CEBPB, CEBPD, transcriptome, gene enrichment, apoptosis, cell cycle, dependency genes, tumor suppressors

## Abstract

**Simple Summary:**

Dpep is a novel cell-penetrating peptide that selectively promotes the death of multiple tumor cell types in vitro and in vivo, and that is a potential therapeutic option for cancer. To better understand how it kills cancer cells, we compared gene expression levels in each of six diverse cancer cell lines before and after peptide treatment. An analysis of the data suggested a mechanism in which Dpep initiates a cascade of perturbations in gene expression that are particular to each cancer cell type, but that represent shared pathways that regulate cell growth and survival. These findings provide insight as to how drugs such as Dpep are able to impact the survival of a wide variety of tumor cell types, and identify how it might be combined with other cancer drugs for optimal efficacy.

**Abstract:**

Dpep is a cell-penetrating peptide targeting transcription factors ATF5, CEBPB, and CEBPD, and that selectively promotes the apoptotic death of multiple tumor cell types in vitro and in vivo. As such, it is a potential therapeutic. To better understand its mechanism of action, we used PLATE-seq to compare the transcriptomes of six cancer cell lines of diverse origins before and after Dpep exposure. This revealed a context-dependent pattern of regulated genes that was unique to each line, but that exhibited a number of elements that were shared with other lines. This included the upregulation of pro-apoptotic genes and tumor suppressors as well as the enrichment of genes associated with responses to hypoxia and interferons. Downregulated transcripts included oncogenes and dependency genes, as well as enriched genes associated with different phases of the cell cycle and with DNA repair. In each case, such changes have the potential to lie upstream of apoptotic cell death. We also detected the regulation of unique as well as shared sets of transcription factors in each line, suggesting that Dpep may initiate a cascade of transcriptional responses that culminate in cancer cell death. Such death thus appears to reflect context-dependent, yet shared, disruption of multiple cellular pathways as well as of individual survival-relevant genes.

## 1. Introduction

The transcription factors ATF5 (activating transcription factor 5), CEBPB (CCAAT enhancer binding protein beta), and CEBPD (CCAAT enhancer binding protein delta) play key roles in the formation, growth, survival, metastasis, and treatment resistance of a wide variety of solid tumors [1]. To bind DNA and become transcriptionally active, these factors must form specific homo- and heterodimers via their basic leucine zipper domains [2]. To selectively target these factors, we designed “dominant negative” decoy peptides that bind their leucine zippers, but that lack the capacity to associate with DNA, thus interfering with their transcriptional activities [1,3,4,5]. Additionally, to permit the decoy peptides to pass through tissue barriers and into cells, we fused them with an N-terminal “penetratin” domain [6]. One set of such peptides, containing a portion of the ATF5 leucine zipper, appears to selectively associate with CEBPB and CEBPD, but not with ATF5 itself [7,8]. A second set, containing portions of the CEBPB and CEBPD leucine zippers and designated as Bpep and Dpep, respectively, appear to selectively target not only CEBPB and CEBPD, but also ATF5 [5,8]. Both sets of peptides promote apoptotic death of a wide variety of tumor cells, both in culture and in animal models [5,9]. In contrast, they do not cause the death or inhibit the growth of non-transformed cells, and appear to have no detectable side effects or promote the death of normal cells in multiple rodent models [5,7,9]. These properties support the potential use of these and related peptides as therapeutic agents [1]. In this context, it is, therefore, important to understand their mechanisms of action.

The mechanisms by which the peptides cause the death of malignant cells are only partially understood. A variety of approaches have shown such death to be apoptotic [5,9]. Critical features of peptide-promoted death found thus far in all cells treated with the peptides include depletion of the survival protein SURVIVIN (product of the *BIRC5* gene) and elevation of the death gene *BMF* (BCL2-modifying factor) [5,10]. In some cell lines, there is also appreciable downregulation of the BCL2 (B-cell lymphoma 2) protein [5]. Of potential relevance, *BCL2* is reported to be a direct transcriptional target of ATF5 [11].

However, beyond these findings, there is little evidence as to what may lie between target engagement by the peptides and activation of distal apoptotic pathways. Because the peptides target transcription factors, we hypothesized that their mechanism of action would include perturbation of the transcriptional landscape, which, in turn, would ultimately trigger elements of the apoptotic machinery. To approach this issue, we investigated and compared the transcriptomes of six different tumor cell lines of diverse origins before and after Dpep treatment. The findings presented herein indicate that the peptide initiates multiple context-dependent transcriptional responses. While the patterns of the responses are cell-specific, they include many alterations of individual gene expression and of functional pathways that are shared among different types of cancer cells and that lie upstream of apoptotic signaling. The data also suggest that such events are triggered by cascades of transcription factor activity.

## 2. Materials and Methods

### 2.1. Cell Culture and Cell Lines

T98G, MDA-MB-231, MCF7, A549, HCT116, and A375 cells were obtained from and authenticated by the ATCC (Manassas, VA, USA). Cells were cultured prior to the experiments in DMEM supplemented with 10% fetal bovine serum. All cultures were maintained at 37 °C in a humidified incubator in an atmosphere containing 5% CO_2_. All lines were tested and found to be negative for mycoplasma (ATCC Universal Mycoplasma Detection Kit).

### 2.2. Dpep

Dpep was purchased as an acetate salt from Alan Scientific (Gaithersburg, MD, USA) and dissolved in 10% glycerol in PBS, pH 7.2, then stored as 2 mM aliquots at −80 °C prior to final dilution for the experiments. The Dpep sequence has been described previously [5]. All cells were treated with the same batch of peptides. Prior testing established that the peptide used for these studies had efficacy and potency on each cell line at concentrations similar to those reported previously [5].

### 2.3. PLATE-Seq

For the PLATE-seq analysis [12], cells were seeded onto 96-well culture plates in 0.2 mL of DMEM supplemented with 10% FBS. The plates were pre-coated with polylysine (0.1 µg/µL) overnight and dried. There were six replicates per condition for each cell line. To assure the uniformity of the cultures, 1–2 lines were processed at a time. After 24 h, the medium was replaced with DMEM supplemented with 2% FBS with or without 20 µM Dpep. Cell confluency was at least 70% at this time. After 48 h, the culture medium was removed and the wells were washed twice with PBS. The cells were then dissolved in 20 µL/well of TCL lysis buffer (Qiagen, Germantown, MD, USA) containing 1% freshly added 2-mercaptoethanol. The plates were then shaken for 5 min on a rotary shaker and centrifuged at 3000 rpm for 1 min. The plates were then sealed, quick-frozen on dry ice, and stored at −80 °C until use. When all lines had been treated and harvested in this way, the samples were thawed and transferred to mRNA TurboCapture plates (Qiagen) and then processed by PLATE-seq as described in detail elsewhere [12]. The total counts per individual sample ranged from 0.8 to 4.7 × 10^6^ and averaged 2.3 ± 0.1 × 10^6^.

### 2.4. Data Analysis

Distribution testing of the expression data using quantro analysis showed significant differences in the shapes of the gene expression distributions of the samples, hence indicating the use of smooth quantile normalization. Multidimensional scaling, principal component analysis, and hierarchical clustering confirmed that the expression data for the different cell lines were segregated from one another, and that in each case, there was also segregation between Dpep-treated and untreated cells. For each line, differential gene expression for the untreated and Dpep-treated cells was determined using edgeR and included quasi-negative binomial error estimation, as well as robust estimation of both the dispersion and the model fit to reduce the effect of outliers. Significance was set at a cutoff of a Benjamini–Hochberg false discovery rate (FDR) of <0.05. All raw and processed data associated with this study are available at the Gene Expression Omnibus under accession number GSE244579.

### 2.5. Bioinformatic Analysis

Over-representation analysis (ORA) and gene set enrichment analysis (GSEA) were carried out online (https://www.webgestalt.org/, accessed June to September 2023) using WebGestalt (WEB-based GEne SeT AnaLysis Toolkit) 2019 [13], and we accessed both the gene ontology (Biological process noRedundant; Molecular Function noRedundant) and pathway (KEGG and Reactome) databases [13,14,15,16,17].

Cell-line-specific dependency genes and common essential genes [18] were identified using the DepMap portal (https://depmap.org/portal/, accessed July 2023) and the Public 23Q2 database (accessed July 2023).

Transcription factor enrichment analysis (TFEA) was performed online (https://maayanlab.cloud/chea3/, accessed July to September 2023) using ChEA3 (ChIP-X Enrichment Analysis Version), an enrichment tool based on “orthogonal omics integration” [19].

## 3. Results

### 3.1. Experimental Scheme

**Figure 1:**
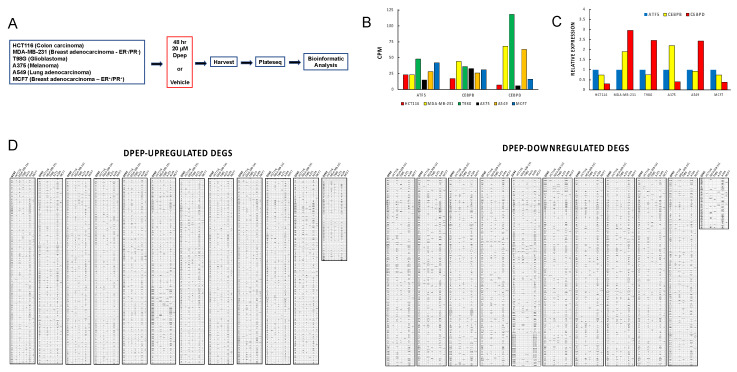
PLATE-seq transcriptome analysis of 6 cancer cell lines before and after treatment with Dpep. (**A**) Experimental scheme. (**B**) Levels of *ATF5*, *CEBPB*, and *CEBPD* transcripts in 6 cancer cell lines without Dpep treatment. Data are given as counts per million reads (CPM). (**C**) Relative expression of *ATF5*, *CEBPB*, and *CEBPD* transcripts in 6 cancer cell lines (without Dpep). Data are normalized to *ATF5*. (**D**) Comparison of significance (FDR ≤ 0.05) Dpep-upregulated and -downregulated DEGs in all 6 cancer cell lines.

To illuminate the potential mechanisms by which Dpep promotes the death of a wide range of tumor cell types, we carried out PLATE-seq (a technology that permits high-throughput transcriptomic analysis) of multiple samples [12]), on six diverse human tumor cell lines: HCT116 (colorectal carcinoma), MDA-MB-231 (triple-negative breast cancer), MCF7 (breast cancer), T98G (glioblastoma), A375 (BRAF mutated melanoma), and A549 (non-small-cell lung cancer). In each case, the cells were treated with or without 20 µM Dpep for 48 h prior to analysis (Figure 1A). This Dpep concentration was near the IC_50_ for each line, and under these treatment conditions, appreciable cell death only became apparent by about 72 h, with little at 48 h [5].

### 3.2. ATF5, CEBPB, and CEBPD Are Differentially Expressed in Each Cell Line

Because Dpep is designed to target ATF5, CEBPB, and CEBPD, we examined and compared the expression of these transcription factors in each cell line. The data confirmed that each line has detectable levels of transcripts encoding all three factors before Dpep exposure. However, there were significant differences in expression between and within the various lines (Figure 1B,C). Most conspicuously, when expressed as counts per million reads, *CEBPD* was considerably more highly expressed in MDA-MB-231, T98G, and A549 cells compared with the other three lines (Figure 1B). When the expression in each line was normalized to that of ATF5 (Figure 1C), *CEBPD* was also the most highly expressed in these three lines, and was most abundant in A375 cells.

### 3.3. Dpep Elicits Context-Dependent Changes in Gene Expression

As anticipated, Dpep promoted numerous changes in gene expression. The numbers of differentially expressed genes (DEGs; FDR ≤ 0.05) varied between lines. A549 (3350 DEGs) and MDA-MB-231 cells (3194 DEGs) showed the greatest number of changes; T98G cells (1621 DEGs) an intermediate number; and MCF7 (711 DEGs), HCT116 (638 DEGs), and A375 (517 DEGs) the fewest. A comparison of the individual DEGs elicited by Dpep in each line revealed distinct, line-dependent patterns of changes in both upregulated and downregulated DEGs (Figure 1D). This likely reflects the cellular background and genetic landscape of each line.

### 3.4. A Cohort of Genes Is Regulated across Multiple Cell Lines

**Figure 2:**
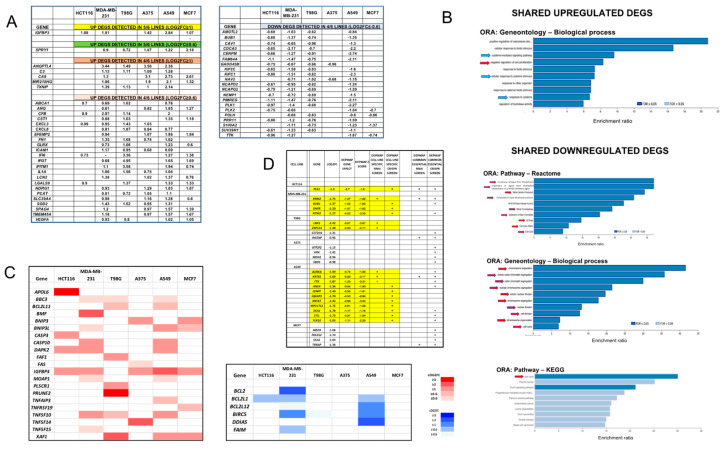
Dpep promotes both context-dependent and shared DEG responses, including those encoding genes involved in apoptosis and dependency. (**A**) Dpep-regulated DEGs shared across multiple cell lines. (**B**) Enrichment analysis of shared upregulated and downregulated DEGs. Blue arrows indicate categories relevant to cytokine signaling; red arrows indicate categories relevant to regulation of the cell cycle. (**C**) Dpep regulates both context-dependent and shared DEGs involved in apoptosis. (**D**) Dpep downregulates genes identified as cell-line-specific “dependency” genes (highlighted in yellow) and those identified as “common essential” on the DepMap portal.

While each line showed different patterns of regulated genes, about half of the DEGs for each line were shared with at least one other line (Figure S1). We further queried whether there were specific DEGs shared among the various lines (Figure 2A; Supplementary Materials Spreadsheets S1 and S2). No DEGs were shared among all six lines. An examination of DEGS with a cutoff of Log_2_FC ≥ 1 or ≤−1 yielded a single gene, *IGFBP3*, that was upregulated in 5/6 lines. Although *IGFBP3* is expressed by T98G cells, it was not regulated there by Dpep. IGFBP3 has been described as having tumor-suppressive activity in a variety of neoplasias via pleiotropic actions [20,21]. Of particular relevance, it is reported to promote apoptosis and/or suppress proliferation of MCF7 [22,23], MDA-MB-231 [24], and A549 [25,26] cells. IGFBP3 has also been associated with oncogenic actions in some contexts [27], including GBM [28,29].

When the cutoff was set to Log_2_FC ≥ 0.6 or ≤−0.6, a second gene, *SPRY1*, emerged as upregulated in five lines (but not in HCT116 cells). SPRY1 is described to have context-dependent tumor-suppressing or tumor-promoting activity and is reported to suppress the growth of A549 [30], A375 [31], and MCF7 [32] cells. In the case of MDA-MB-231 cells, one report [33] has indicated that SPRY1 suppresses growth, while another [34] indicates promotion of growth.

In addition to the above, there were also DEGs common to four of the six lines. With a cutoff of Log_2_FC ≥ 1 or ≤−1, five upregulated DEGs were common to four lines: *CA9*, *MIR210HG*, *ANGPTL4*, *C3*, and *TXNIP*. Of these, TXNIP (thioredoxin-interacting protein) upregulation in particular has been associated with cancer cell death [35]. Moving the cutoff to Log_2_FC ≥ 0.6 or ≤−0.6, the total number of upregulated DEGs shared by at least four of six lines reached 28, and the number of shared downregulated DEGS was 21 (Figure 2A).

To determine whether the DEGs shared across at least four cell lines also shared common functions, we carried out over-representation analyses (ORA) separately for these upregulated and downregulated DEGS (Log_2_FC ≥ 0.6 or ≤−0.6) (Figure 2B). A gene ontology assessment for biological processes identified 11/30 upregulated DEGs shared by at least four lines as members of a “cytokine-mediated signaling pathway”. The next two categories were “cellular response to cytokine stimulus” and “response to cytokine”, with similar lists of DEGs (Figure 2B). A pathway analysis via Reactome also returned “signaling by interleukins”. These results likely reflect the upregulation by Dpep of numerous DEGs encoding cytokines and interleukins, as well as interferon-regulated genes (see below).

With regard to downregulated DEGs shared by four lines, gene ontology (biological process) and pathway (KEGG and Reactome) analyses identified significant enrichment of multiple categories related to the cell cycle and mitosis (Figure 2B). This complements the finding of enrichment for upregulated shared DEGs categorized under “negative regulation of cell proliferation”. These findings mirror the more general observation (see below) that Dpep negatively regulates multiple cell cycle genes in most of the lines which we have assessed.

### 3.5. Context-Dependent Regulation of Pro- and Anti-Apoptotic Genes

Prior studies have shown that Dpep promotes the apoptotic death of transformed cells [5]. In all tested lines, this was correlated with downregulation of the survival protein SURVIVIN (product of the *BIRC5* gene) and with upregulation of pro-apoptotic *BMF* (BCL2-modifying factor). The BCL2 protein was also downregulated to a variable degree, depending on the cell line [5]. Although our analysis did not show enrichment in apoptotic DEGS, we identified a number of Dpep-regulated genes associated with apoptotic cell death (Figure 2C). As with other DEGs, those associated with apoptosis manifested a context-dependent pattern of response. Each line showed upregulation by >twofold of at least three pro-apoptotic genes: HCT116 (*APOL6*, *CASP3*, *DAPK2*, *IGFBP3*); MDA-MB-231 (*BMF*, *CASP10*, *DAPK2*, *IGFBP3*, *TNFSF10*); T98G (*FAF1*, *PLSCR1*, *PRUNE2*, *XAF1*); A375 (*BNIP3*, *IGFBP3*, *TNFSF14*); A549 (*BNIPL3*, *CASP10*, *DAPK2*, *IGFBP3*, *TNFSF10*, *XAF1*); and MCF7 (*BNIP3*, *IGFBP3*, *XAF1*)**.** Interestingly, these upregulated DEGs represent both the intrinsic and extrinsic apoptotic mechanisms. Although each line displayed a distinct set of upregulated pro-apoptotic genes, several were upregulated by greater than twofold in three different lines: *DAPK2* (HCT116, MDA-MB-231, A549) and *XAF1* (T98G, A549, MCF7), and, in one case (*IGFBP3*), in five of the six lines.

Regarding the downregulated pro-survival genes, there were relatively few, with none regulated by >twofold for HCT116, T98G, A375, or MCF7 cells (Figure 2C). It is of interest that *BCL2* was downregulated by about fourfold in MDA-MB-231 cells, a line that showed strong downregulation of the BCL2 protein by Dpep [5]. *BIRC5* (encoding SURVIVIN) was also downregulated by >twofold in MDA-MB-231 and A549 cells. A past study showed that diminished stability also contributes to a loss of the SURVIVIN protein in peptide-treated tumor cells [10]. Finally, the survival gene *BCL2L12* was downregulated by >twofold in A549 cells. Taken together, these observations show that Dpep regulates multiple apoptotic genes and does so in a context-dependent, yet partially overlapping, manner.

### 3.6. Dpep Downregulates Dependency Genes

To identify single downregulated genes that could account for the inhibitory effects of Dpep on tumor cell growth and survival, we queried DepMap (Public 23Q2) to uncover candidates specific to each cell line studied herein. In particular, we searched for DEGs in each line in which the log_2_FC was ≤−0.9 and for which the DepMap gene effect was ≤−0.5 and the Z score was ≤−1.6 according to either siRNA or CRISPR screens. As observed with other comparisons of DEGs described herein, the specific dependency genes regulated by Dpep in each line were distinct (Figure 2D). The number of such dependency DEGS varied among lines, ranging from 0 (A375, MCF7) to 1 (HCT116), 2 (T98G), 4 (MDA-MB-231), and 11 (A549). Notably, many of such genes regulated by Dpep (i.e., *PLK1*, *RRM2*, *BUB1*, *AURKA*, *ANLN*, *MKI67*, *SKA1*) encode proteins required for successful cell cycle completion. For the lines with <4 dependency DEGs, we also asked whether there were additional “common essential” genes identified in DepMap that were downregulated in response to Dpep. As listed in Figure 2D, we identified several candidates in A375, MCF7, and T98G cells.

### 3.7. Dpep Downregulates Cell-Cycle-Related Genes in Multiple Lines

**Figure 3:**
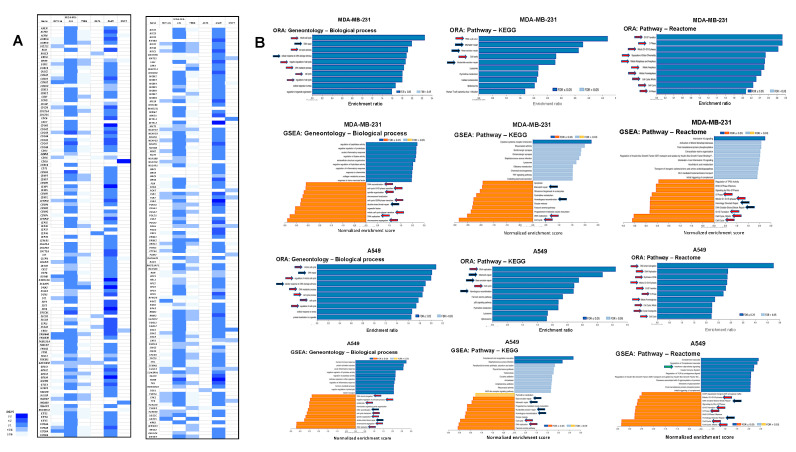
Dpep downregulates numerous DEGs encoding cell cycle genes. (**A**) Downregulation of DEGs encoding cell cycle genes in 6 cancer cell lines. (**B**) ORA and GSEA gene ontology and pathway analyses of total DEGs for MDA-MB-231 and A549 cells showed significant enrichment of DEGs related to various cell cycle events and DNA repair. Red arrows indicate categories relevant to various phases of the cell cycle. Black arrows indicate categories relevant to DNA repair.

Apoptotic cell death is regulated by a variety of both transcriptional and non-transcriptional mechanisms. We, therefore, looked for upstream transcriptional changes that might affect the balance between pro- and anti-apoptotic components. Numerous studies have established a link between apoptosis and disruption of the activity/expression of cell cycle components [36,37,38]. The above observations, as well as analyses of individual cell line DEGs, indicate that Dpep causes widespread downregulation of cell-cycle-related genes in multiple lines. The effects on genes associated with cell cycle progression, DNA synthesis, and mitosis are shown in Figure 3A. In terms of absolute numbers of cycle-related DEGs and fold downregulation, MDA-MB-231 and A549 cells showed the most evident responses. In both lines, nearly 6% of the total DEGs could be assigned to this category. Despite their differing tumor origins, 89% of the cycle-related DEGs downregulated in MDA-MB-231 cells were also downregulated in A549 cells. Genes which were particularly affected in both lines (down > fourfold) included *CDCA3*, *CDCA7*, *ERCC6L*, and *TICRR*. For each of these genes, knockdown was reported to suppress proliferation and promote apoptosis in malignant lines, including lung and breast cancers [39,40,41,42]. In support of these observations, ORA and GSEA gene ontology and pathway analyses of the total DEG PLATE-seq data for both MDA-MD-231 and A549 cells showed significant enrichment of DEGs related to various cell cycle events, including G1/S transition, DNA replication, S phase, and mitosis (Figure 3B).

HCT116 and T98G cells also exhibited numerous DEGs associated with the cell cycle, representing 12% and 7% of total DEGs in each line, respectively. In these lines, also, ORA and GSEA analyses identified significant enrichment of regulated genes associated with various phases of the cell cycle (Figure S2). Interestingly, a high proportion of cycle-related DEGs in the two lines (86 and 96%, respectively) were also shared with A549 cells. Among the down-regulated cycle-related DEGs in HCT116 cells were *FAM83D* (down by 2.4-fold) and *RGCC/RGC32* (down by 4-fold), knockdown of which was reported to promote tumor cell apoptosis [43,44]. Downregulated *MAD2L1* in T98G cells was also reported to cause cancer cell apoptosis when knocked down [45].

Thus, in four of six of the assessed lines, Dpep appeared to disrupt the expression of numerous genes involved in multiple steps of cell replication. Given the association between dysregulated cell cycle and apoptotic death, such effects are likely to contribute to Dpep’s inhibition of tumor cell growth and survival in these and, likely, additional cancer cell types. In contrast, A375 and MCF7 cells showed less robust regulation of cell-cycle genes, with only 1.5% and 2.7% of DEGs, respectively, assigned to this activity; neither ORA nor GSEA analysis revealed enrichment in this category. Nevertheless, it may be relevant that in MCF7 cells, the replication-related kinase gene *CDK8* was downregulated by 30-fold in response to Dpep, and that CDK8 inhibition or knockdown promotes cancer cell apoptosis [46,47].

### 3.8. Downregulation of DEGs Associated with DNA Repair

In addition to cell-cycle-related DEGs, two lines, MDA-MB-231 and A549, exhibited conspicuous downregulation of numerous DEGs associated with DNA repair (Figure S3). ORA and GSEA analyses confirmed their enrichment in both DEG sets (Figure 3B). While many of the DEGs in this category overlapped with those involved in cell cycle regulation, a number, such as *BARD1*, *BRCA1*, *CHEK1*, *FANCB*, *FANCC*, *NEIL3*, *RAD51*, *TONSL*, and *XRCC3*, were mainly associated with DNA repair. Such downregulation may tip the balance towards apoptosis, and supports the potential use of a combination of Dpep and DNA-damaging agents in certain cancers.

### 3.9. Selective Induction of DEGs Associated with Interferon Responses

**Figure 4:**
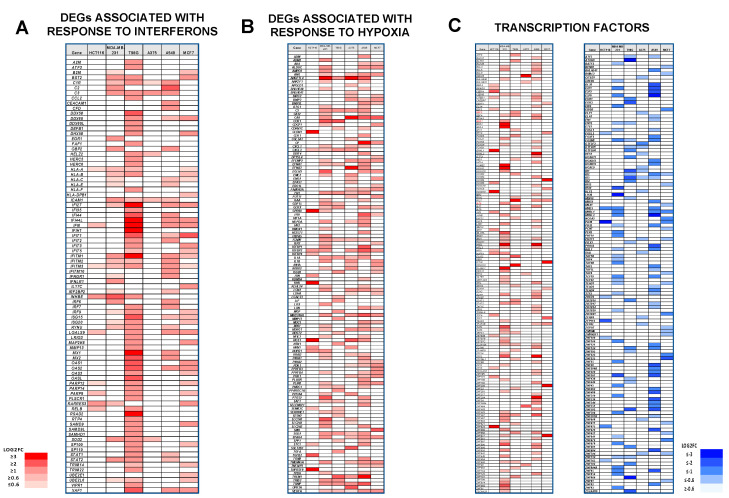
(**A**). Dpep upregulated genes associated with responses to interferons. (**B**). Dpep upregulates genes associated with responses to hypoxia. (**C**). Dpep regulates multiple transcription factors. DEGs in each category were compared across 6 cell lines.

An analysis of Dpep-responsive DEGs revealed the unanticipated upregulation of interferon-responsive genes in all six lines (Figure 4A). In particular, this was especially evident in T98G and MCF7 cells, for which both the ORA and GSEA analyses identified significant enrichment in interferon response DEGs (Figures S2 and S4). Examples include *IFI27*, *IFI44L*, *IFIT1*, *OAS1*, and *OAS2*. Notably, each of these genes has been associated with the promotion of cancer cell apoptosis [48,49,50,51,52]. In both of these cell lines, 4–5% of the total DEGs were included in this category. In contrast, approximately 1% of the total DEGs were associated with interferon responses in the remaining lines.

### 3.10. Dpep Upregulates Genes Indicative of a Response to Hypoxia

Examination of Dpep-affected transcripts revealed upregulation in all lines of DEGs that have been described as hypoxia-responsive (Figure 4B). While each line had a unique pattern of regulated hypoxia-responsive genes, a number of such DEGs were shared by at least three of the lines, including, for example, *ANGPTL4*, *CA9*, *CXCL3*, *EGLN3*, *IGFBP3*, *MIR210HG*, *NDRG1*, *SOD2*, *TREM1*, and *TXNIP*. An ORA gene ontology analysis of biological processes indicated significant enrichment in the categories “response to hypoxia” and “response to oxygen levels” in A375 cells, and in “response to oxygen levels” in MCF7 cells (Figure S4). In consonance with this, the percentages of total DEGs identified as hypoxia-responsive were 8.9% and 5.8% in A375 and MCF7 cells, respectively, and between 2 and 3% in the remaining lines. One possibility is that such upregulation reflects an oxidative stress response, and that this, in turn, contributes to elevated apoptosis. As noted above, *IGFBP3*, which was upregulated in five of six lines, is able to promote tumor cell death, as is *TXNIP*, which was upregulated in four lines.

### 3.11. Downregulation of Oncogenes and Upregulation of Tumor Suppressors in Response to Dpep

Additional scrutiny of the PLATE-seq data revealed cell-line-specific patterns of regulated DEGs encoding proteins described in multiple studies as either oncogenes or tumor suppressors (Figure S5). In each case, the oncogenes were downregulated and the tumor suppressors were upregulated. Downregulated oncogenes represented 2–3% of the total DEGs in all lines except MCF7 (0.7% of total). Each line had at least one oncogene that was downregulated by 6–30-fold. For example, *MERTK*, implicated as an oncogene in melanoma and other cancers [53,54], was downregulated in A375 (log_2_FC = −3.58) and A549 cells ((log_2_FC = −2.08), while *PLCE1*, identified as an oncogene overexpressed in a variety of cancers [55,56], was downregulated in MDA-MB-231 (log_2_FC = −1.98), MCF7 (log_2_FC = −4.31) and A549 cells (log_2_FC = −0.97). As is emblematic of many of the regulated oncogenes, knockdown of *MERTK* or *PLCE1* has been reported to trigger cancer cell apoptosis [53,54,55,56]. Although each line had a unique set of responsive oncogenes, as in overall DEGs, MDA-MB-231 and A549 had a striking similarity in terms of shared DEGs in this category (69/90).

Regarding DEGs encoding tumor suppressors, these were upregulated in all lines, with at least one in each line elevated by ≥2.7-fold. While each line had a distinct pattern of upregulated DEGs encoding tumor suppressors, several of such DEGs were upregulated in multiple lines, including the aforementioned *IGFBP3* (five lines); *TXNIP* and *NDRG1* (four lines); and *RAB37* and *XAF1* (three lines). The upregulation of such genes by Dpep is anticipated to negatively influence growth and survival.

### 3.12. Dpep Affects the Expression of Numerous Transcription Factors

Given the varied and context-dependent effects of Dpep on the transcriptome, we considered how it might affect the expression of transcription factors. Exploration of the PLATE-seq data revealed cell-specific patterns of multiple up- and downregulated DEGs encoding proteins that regulate transcription (Figure 4C). In each case, the proportion of such DEGs vs. total DEGs was 3–5%. All six lines had at least one such DEG upregulated by at least eightfold: HCT116 (*HOXD10*, *ZNF528*); MDA-MB-231 (*EGR2*, *EGR4*, *HOMEZ*, *HOXA1*, *NFE2*, *PLAG1*); T98G (*PKNOX2*, *IF27*); A375 (*BEND5*); A549 (*GRHL2*, *NFE2*, *ZFPZ*, *ZNF284*); and MCF7 (*ZNF577*, *ZNF208*, *PITX2*, *HOXB6*). Four of the six lines also showed at least one transcription factor downregulated by at least eightfold: HCT116 (*PAX9*); MDA-MB-231 (*LHX6*); T98G (*ATOH8*); and A549 (*E2F8*, *MYBL2*). While each line showed a distinct pattern of regulated transcription factors, there were several instances in which specific transcription factors were similarly responsive across multiple lines. These were *IF27* (up in T98G, A549, and MCF7 cells); *ZNF467* (up in MDA-MB-231, T98G, and A549 cells); *FOXM1* (down in MDA-MB-231, T98G, and A549 cells); *ID2* (down in T98G, A549, and MCF7 cells); *ID3* (down in T98G, A375, and A549 cells); *MDX3* (down in HCT116, MDA-MB-231, and A549 cells); and *MYBL1* (down in MDA-MB-231, A549, and MCF7 cells). Taken together, these observations indicate that Dpep triggers dysregulated expression of multiple transcription factors in a context-dependent fashion. The presence of multiple regulated TFs suggests a cascade that is initiated by interference with ATF5, CEBPB, and CEBPD activities. This, in turn, may account in part for the large numbers of cell-type-specific DEGs elicited by Dpep treatment, and, ultimately, for the effects of the peptide on cancer cell growth and survival.

### 3.13. Transcription Factor Enrichment Analysis (TFEA) Indicates Shared and Cascading Transcriptional Pathways

**Figure 5:**
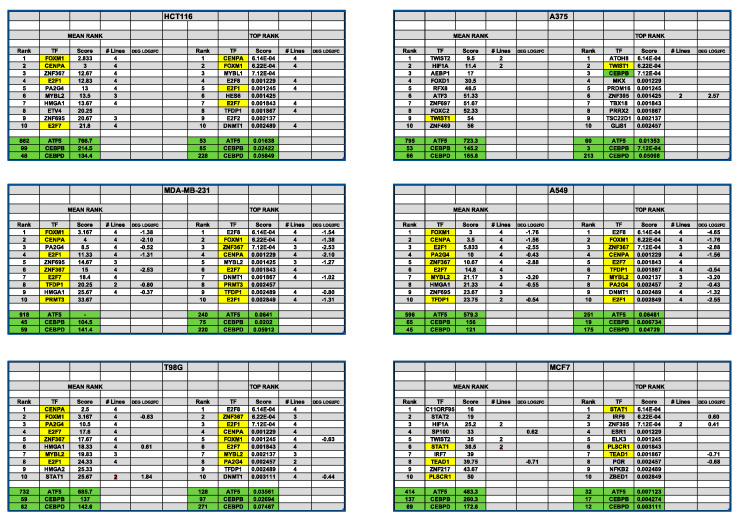
ChEA3 analysis of DEGs induced by Dpep in six cancer cell lines. Transcription factor enrichment analysis (TFEA) was carried out by ChEA3 on total DEGs in each cell line. The top 10 ranking results from both the Top Rank and Mean Rank libraries are shown for each line. For each cell line, the ranks and scores are given for each TF, as well as the numbers of lines in which the specific TF is ranked in the same library. If the TF is also a DEG in the given cell line, its degree of regulation (as log_2_FC) is also given. For each line, TFs that appear in both libraries are highlighted in yellow. Ranks and scores for ATF5, CEBPB, and CEBPD are highlighted in green.

To further probe the regulatory networks of transcription factors that might contribute to the responses of cancer cells to Dpep, we carried out transcription factor enrichment analysis (TFEA) using ChEA3, an enrichment tool based on “orthogonal omics integration” [19]. The analyses were carried out using total DEGs for each of the six cell lines. ChEA3 provides two outputs, the Mean Rank and Top Rank libraries. We focused on the top 10 ranked TFs in each library and, in particular, on those that appeared in both rankings. We also looked for highly ranked TFs that were common to multiple cell lines and whether they were regulated in response to Dpep. An examination of the analyses (Figure 5) revealed that four lines, i.e., HCT116, MDA-MB-231, T98G, and A549, showed strikingly similar lists of enriched TFs. This was the case for both the comparison of the Mean Rank and Top Rank results for each cell line and for the comparison of such results among the four lines. Particular TFs that stood out included FOXM1, CENPA, E2F1, E2F7, ZNF367, PA2G4, and MYBL2. It is notable that the four lines in which they were identified as regulators of DEGs were also the lines that showed significant enrichment of downregulated cell cycle genes. Moreover, while the identified TFs were not regulated by Dpep in all four lines, where they did appear as DEGs (particularly in MDA-MB-231 and A549 cells), they were invariably downregulated.

As is the case for other aspects discussed herein, the ChEA3 analyses of A375 and MCF7 DEGs differed from those in the other four lines, and the scores tended to be less robust. This underscores the context-dependent nature of the response to Dpep. One intriguing observation was the appearance of HIF1A (hypoxia-inducible factor 1 subunit alpha) in the sets of lists for both cell lines, which may potentially be relevant to their enrichment for hypoxia-related DEGs, as discussed above.

Another notable observation from the ChEA3 analysis was the inconsistent rankings of CEBPD, CEBPB, and ATF5 and their generally low scores. This suggests the interpretation that interference with their activities triggers a largely indirect cascade of downstream alterations in TF activities that ultimately accounts for the patterns of the observed DEGs. Consistent with this are the large numbers of Dpep-responsive DEGS that encode TFs, as described above.

While the ChEA3 analysis did not provide consistent rankings for ATF5, CEBPB, or CEBPD when total DEGs were considered, we observed that such an analysis of only upregulated DEGs for each line returned enrichment scores in which at least one of the three factors was in the top 50 TFs in both the Mean and Top Rank libraries (Figure S6). In addition, for the upregulated DEGs, there was a reasonable concordance between the two libraries for the rankings of CEBPB and CEBPD, as well as among those for various lines. Among the TFs that were recurrent in the various libraries (in addition to CEBPB and CEBPD) were IRF1, BLHHE40, GLMP, BATF2, STAT2, STAT3, PLSCR1, ATF3, RELB, JUN, and JUNB. These observations support the idea that Dpep initiates a cascade of changes in cellular transcriptional activity that is context-dependent, yet involves an overlapping set of TFs. While some of these TFs were regulated in response to Dpep (in most cases upwards) in some lines, this was not the case in all lines. Thus, the cascade may also move by transcription-independent mechanisms such as post-translational modifications and altered stability.

**Figure 6:**
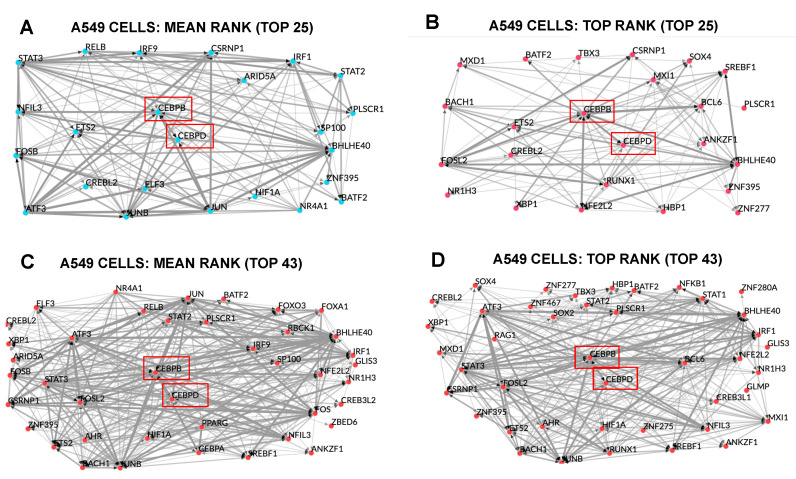
TF co-regulatory network, as determined by ChEA3 using the top 25 (**A**,**C**) and 43 (**B**,**D**) TFs in the indicated Mean Rank and Top Rank libraries for upregulated DEGs in A549 cells. Positions of CEBPB and CEBPD are indicated by red boxes.

To conceptualize potential networks through which Dpep might generate a transcriptional cascade by interfering with CEBPB/CEBPD activity, we used the TF co-regulatory tool in ChEA3 to analyze the top 25 and 43 enriched TFs in both the Mean and Top Rank libraries for upregulated DEGs in A549 cells (Figure 6D). Each analysis included CEBPB and CEBPD and illustrated the complex interactions that can occur between these two TFs, as well as others that may be involved in generating a cascade that ultimately leads to the upregulation of multiple DEGs in this line.

## 4. Discussion

The purpose of this study was to identify and compare the transcriptional responses of multiple tumor cell types to Dpep, a cell-penetrating peptide designed to interfere with ATF5, CEBPB, and CEBPD activity. We also inferred how such changes may lead to the apoptotic death of cancer cells. We achieved this goal using PLATE-seq, a low-cost, genome-wide mRNA profiling methodology which was initially applied for the purpose of screening drug effects across single cell lines [12]. We achieved the highly significant identification of regulated genes across multiple cell lines either exposed or not to the same drug, and were able to accomplish this using frozen samples from lines harvested at different times. We also found that six biological replicates were sufficient to identify DEGs with high significance.

**Figure 7:**
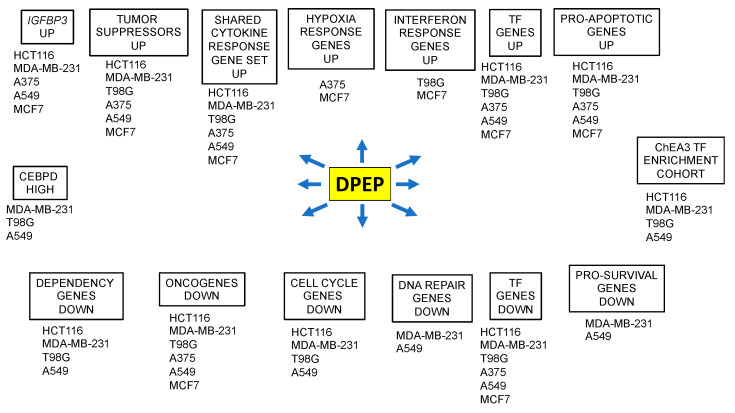
Summary of context-dependent and shared transcriptional responses to Dpep treatment.

The overall finding was a context-dependent pattern of gene regulation that was unique to each of the six assessed cell lines. Within this general diversity, however, there were a number of shared regulated elements and pathways with the potential to negatively affect cell growth and survival (Figure 7). There were also changes in expression in each line of single genes with the potential capacity to disrupt cell growth and survival.

A common endpoint thus far of all cancer lines treated with Dpep has been the onset of apoptotic cell death. At least two pro-apoptotic genes were upregulated by >twofold in each line assessed herein. While each line displayed a unique set of such genes, there was some overlap, with two pro-apoptotic genes, *DAPK2* [57] and *XAF1* [58], upregulated in three of six lines. It was also of interest that the upregulated genes included several, including *TNFSF10* (TRAIL) and *TNFSF14* (LIGHT), that participate in extrinsic apoptotic signaling. Of potential relevance, a past study showed that CP-dn-ATF5, a cell-penetrating peptide that targets CEBPB and CEBPD, sensitizes tumor cells to TRAIL-induced apoptosis [7]. Regarding the downregulation of anti-apoptotic genes, fewer were detected. A notable observation was a > fourfold downregulation of *BCL2* in MDA-MB-231 cells. This correlates with a previously reported strong downregulation of the BCL2 protein by Dpep and Bpep in this line [5]. While many steps in the activation process of apoptotic pathways are non-transcriptional in nature, taken together, these findings indicate that cell-specific regulation of pro- and anti-apoptotic genes likely contributes to the mechanism by which Dpep kills cancer cells.

Analysis of the PLATE-seq data uncovered a number of Dpep-regulated pathways and biological activity groups that can lie upstream of apoptotic activation. Four lines showed substantial downregulation of genes identified by DepMap as being “dependency” genes for those lines. Additionally, we found upregulation of multiple tumor suppressors and downregulation of multiple oncogenes in all lines. Again, while all lines were affected, the patterns of the regulated genes were context-dependent and included genes regulated in up to five of the six lines (*IGFBP3*), as well as those unique to each line.

Other regulated pathways and activities that may lie upstream of apoptosis initiation were shared by subsets of the six lines. Gene ontology analysis identified four lines as having significantly enriched downregulation of genes associated with various steps involved in cell cycle and replication. Interestingly, the two lines not identified as having enrichment for cell-cycle-related genes (A375 and MCF7), were found by gene ontology analysis to show enrichment for hypoxia and interferon responses. These were also the two lines that showed the highest proportions of co-shared DEGS after normalization. As noted above, dysregulations of genes involved in the cell cycle or responsive to hypoxia, or interferons have been described as promoting the activation of apoptotic pathways.

While our analysis has focused on pathways that show disruption by Dpep, it is important to consider that the dysregulation of only a single gene may interfere with cell growth and survival. The examples discussed above include depletion of replication-related transcripts for *CDK8* in Dpep-treated MCF7 cells and the downregulation of “dependency” genes in susceptible cell lines. On the whole, the scenario emerges that in each cancer cell type, Dpep sets in motion multiple streams of altered gene expression that have the potential to culminate in apoptotic death.

How, then, might Dpep initiate such events? Interrogation of the transcriptome data revealed that in all six lines, Dpep regulated numerous transcription factors. While the patterns of the regulated TFs were context-dependent, it is also of interest that several were regulated in three different lines. Among them was downregulated *FOXM1*, a well-studied oncogenic TF with a variety of roles in promoting cancers [59,60], including through the stimulation of cell cycle progression. The regulation of many TFs by Dpep suggested the idea that the peptide triggers a cascade of context-dependent TF activity that ultimately leads to the activation of apoptotic pathways.

Given the design of Dpep, it is anticipated that the induction of such a cascade might start with altered expression of genes directly regulated by ATF5, CEBPB, and CEBPD. Notably, ChEA3 TF enrichment analysis based on total DEGs for each line did not highly rank the three factors, nor were their rankings consistent within or between cell lines. This is consistent with a mechanism in which interference with ATF5/CEBPB/CEBPD activity initiates cascading networks of downstream TFs that culminate in the production of the patterns of DEGs observed herein. The application of ChEA3 analysis to only upregulated DEGs consistently places CEBPB and/or CEBPD in the 50 top-ranked TFs. This enabled us to use the ChEA3 co-regulatory tool to produce a model of how a network of TFs might be interconnected with CEBPB and CEBPD to generate the observed pattern of DEGs in A549 cells.

Regarding the inconsistent ranking of ATF5 in such analyses, this may reflect either its relative lack of involvement in generating DEGs or the present paucity of information regarding its transcriptional targets. For example, one report has indicated that ATF5 (and, thus, potentially its dimerization partners, CEBPB and CEBPD) associates with a novel DNA binding site [61]. In addition, ATF5 is reported to have roles in the mitochondrial stress response [62] and in centrosome function [63], interference with which may also contribute to the generation of DEGs.

Besides directly repressing the transcriptional actions of ATF5, CEBPB, and CEBPD, there are several additional non-mutually-exclusive mechanisms by which Dpep might alter the transcriptomes of cancer cells. For example, the absence of available ATF5, CEBPB, and/or CEBPD may relieve competition for binding to DNA by other TFs, thus permitting an alternate pattern of gene regulation. It also cannot be ruled out that Dpep associates with and serves as an inhibitory decoy for TFs in addition to ATF5, CEBPB, and CEBPD [1]. Finally, as discussed above, as in the case of ATF5, these TFs may also serve non-transcriptional roles, disruption of which by Dpep would affect the cellular transcriptome.

## 5. Conclusions

In conclusion, the transcriptomic data described herein indicate that Dpep promotes the death of a wide range of tumor cells by setting in motion a cascade of TF-driven changes in multiple molecular pathways that converge on apoptosis. These events are context-dependent, yet fall into discrete sets of pathways shared by subsets of tumor cell types. The pathways include upregulation of tumor suppressors; pro-apoptotic genes; and genes responding to hypoxia, cytokines, and interferon. Downregulated pathways include oncogenes; dependency genes; and genes involved in cell cycle regulation, DNA repair, and cell survival.

## Data Availability

All raw and processed Plate-seq data associated with this study are available at the Gene Expression Omnibus under accession number GSE244579.

## Supplementary Materials

The following supporting information can be downloaded at: https://www.mdpi.com/article/10.3390/cancers15225318/s1, Figure S1: Dpep-responsive DEGs show both unique and overlapping patterns of expression in six cancer cell lines; Figure S2: ORA and GSEA gene ontology and pathway analyses of total DEGs for HCT116 and T98G cells shows significant enrichment of DEGs related to various cell cycle events; Figure S3: Dpep downregulates genes associated with DNA repair; Figure S4: Dep regulates genes associated with interferon responses and hypoxia in a subset of cell lines; Figure S5: Dpep upregulates tumor suppressors and downregulates oncogenes with both context-dependent and shared responses in multiple lines; Figure S6: ChEA3 Transcription Factor Enrichment Analysis (TFEA) of upregulated DEGs induced by Dpep in six cancer cell lines; Spreadsheet S1: Shared DEGS log_2_FC > 1, <−1; Spreadsheet S2: Shared DEGS log_2_FC > 0.6, <−0.6.
