# Supplementary material for: Dpep Inhibits Cancer Cell Growth and Survival via Shared and Context-Dependent Transcriptome Perturbations
Source: Cancers (Basel). 2023 Nov 7;15(22):5318. doi: 10.3390/cancers15225318 (PMC10669862; doi:10.3390/cancers15225318)
Supplement: Supplementary file 1 [file cancers-15-05318-s001.zip › cancers-2673388-supplementary figures.pdf]

		HCT116	MDA-MB-231	T98	A375	A549	MCF7
HCT116							
	% NON-OVERLAP	45					
	% OVERLAP		26.8	11.6	2	39.1	5.60
	% OVERLAP NORMALIZED/1000 DEGS		15.1	15.5	8.7	10	13.8
		HCT116	MDA-MB-231	T98	A375	A549	MCF7
MDA-MB-231							
	% NON-OVERLAP		47.2				
	% OVERLAP	4.5		9.4	2.3	44.9	3.7
	% OVERLAP NORMALIZED/1000 DEGS	14.9		12.6	10	11.5	9.3
		HCT116	MDA-MB-231	T98	A375	A549	MCF7
T98G							
	% NON-OVERLAP			48.4			
	% OVERLAP	4.7	22.3		2.9	34	7.1
	% OVERLAP NORMALIZED/1000 DEGS	15.6	12.6		12.7	8.7	17.8
		HCT116	MDA-MB-231	T98	A375	A549	MCF7
A375							
	% NON-OVERLAP				54.1		
	% OVERLAP	2.6	17.9	9.6		31.4	12.7
	% OVERLAP NORMALIZED/1000 DEGS	8.6	10.1	12.9		8	31.8
		HCT116	MDA-MB-231	T98	A375	A549	MCF7
A549							
	% NON-OVERLAP					62.7	
	% OVERLAP	3	20.4	6.5	1.8		2
	% OVERLAP NORMALIZED/1000 DEGS	9.9	11.5	8.7	8		5
		HCT116	MDA-MB-231	T98	A375	A549	MCF7
MCF7							
	% NON-OVERLAP						51.3
	% OVERLAP	4.3	16.5	13.3	7.3	19.8	
	% OVERLAP NORMALIZED/1000 DEGS	14.2	9.3	17.8	31.7	5.04	

Figure S1. Dpep-responsive DEGs show both unique and overlapping patterns of expression in 6 cancer cell lines. Percentages of unique (highlighted in green) and overlapping (highlighted in yellow) Dpep-regulated DEGs among 6 different cancer lines are shown. Overlap comparisons are given based on absolute number and as normalized per 1000 DEGs to take into account the differing numbers of total DEGs observed in each line. Values highlighted in yellow designate the normalized percentages for A375 and MCF7 cells which are substantially higher than those for other cell line pairs.

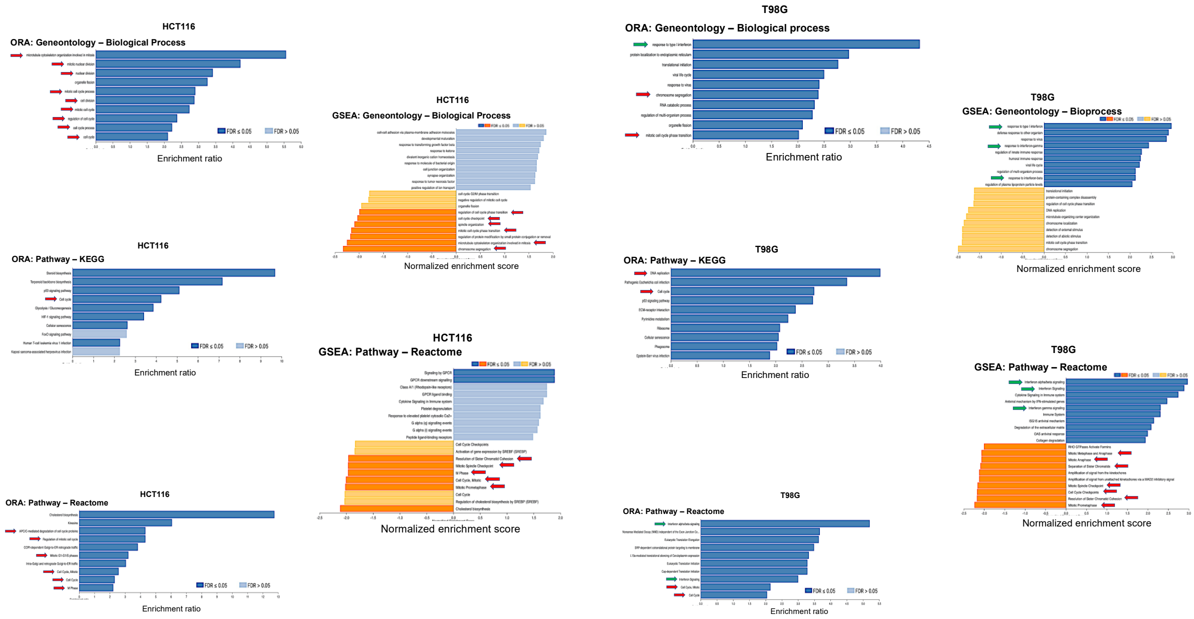

Figure S2. ORA and GSEA geneontology and pathway analyses of total DEGs for HCT116 and T98G cells shows significant enrichment of DEGs related to various cell cycle events. Red arrows indicate categories relevant to various phases of the cell cycle. Green arrows indicate categories relevant to response to interferons.

DEGs ASSOCIATED WITH DNA REPAIR

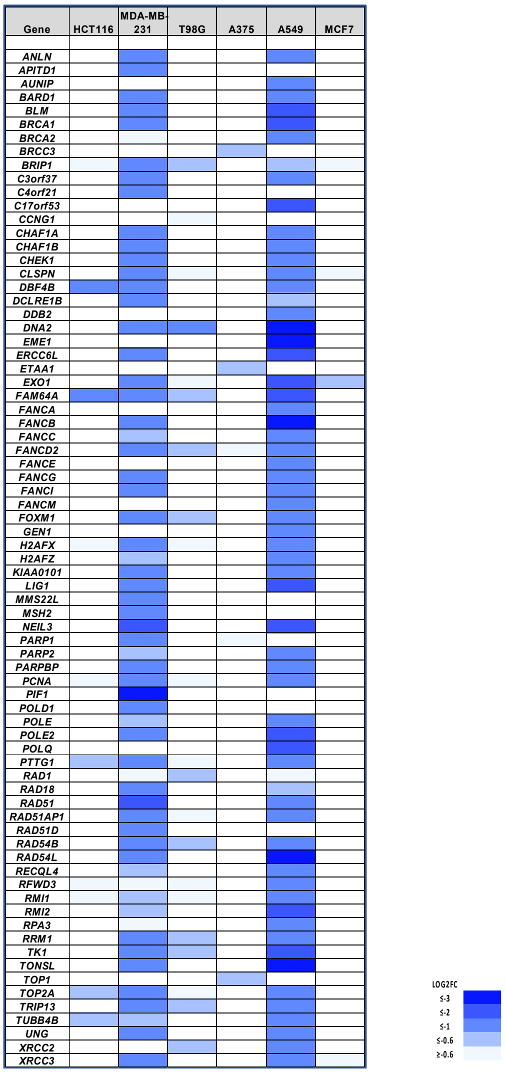

Figure S3. Dpep downregulates genes associated with DNA repair.

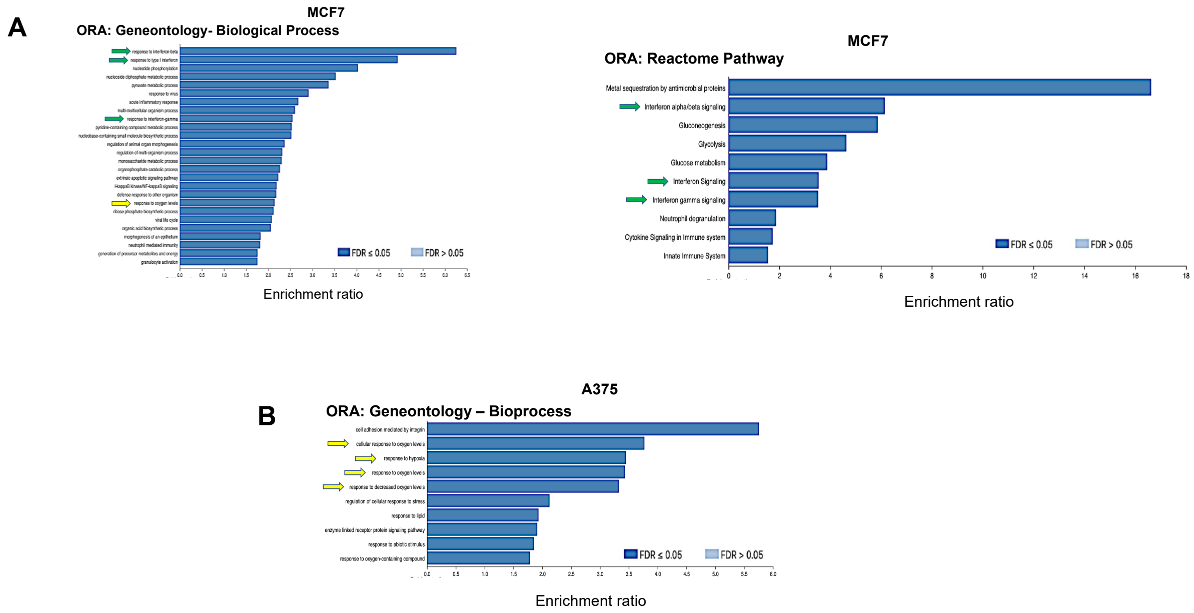

Figure S4. Dep regulates genes associated with interferon responses and hypoxia in a subset of cell lines. A. ORA geneontology and reactome analysis of MCF7 cell total DEGs reveals enrichment of genes associated with responses to interferons (green arrows) and hypoxia (yellow arrows). B. ORA geneontology analysis of A375 total DEGS reveals enrichment of genes associated with hypoxia (yellow arrows).

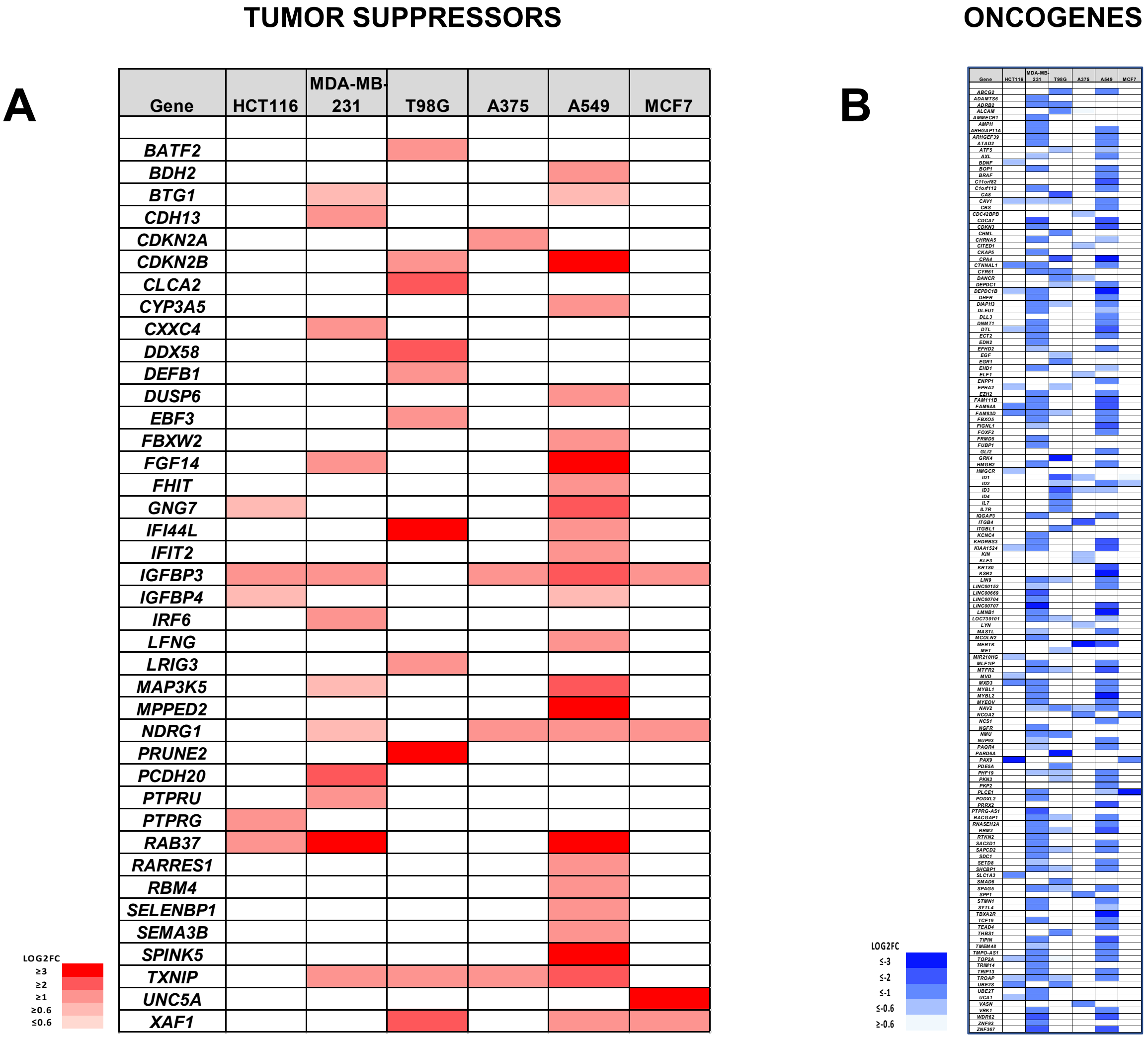

Figure S5. Dpep upregulates tumor suppressors and downregulates oncogenes with both context dependent and shared responses in multiple lines. A. Upregulated tumor suppressors. B. Down regulated oncogenes.

HCT116

	MEAN RANK						TOP RANK	
Rank	TF	Score	# Lines		Rank	TF	Score	# Lines
1	GLMP	7.5	5		1	ETV7	6.14E-04	4
2	BATF2	15.33	4		2	BATF2	6.22E-04	4
3	IRF9	15.75	5		3	IRF9	7.12E-04	3
4	SP100	24.67	3		4	STAT1	0.001229	4
5	IRF1	24.83	6		5	ASH1L	0.001245	
6	STAT2	34.6	4		6	MXD4	0.001425	2
7	SP110	41	2		7	TRAFD1	0.001843	3
8	STAT3	47	5		8	ASCL3	0.001867	2
9	BHLHE40	48	6		9	ZNF385A	0.002137	2
10	TRAFD1	51.33	2		10	IRF7	0.002457	3
11	PLSCR1	53.5	6		11	ZBED1	0.002849	
12	NR1H2	60.5			12	THAP3	0.003071	
13	DDIT3	67.33	2		13	GLMP	0.003111	5
14	KLF6	69.75	3		14	MNT	0.003367	
15	GLIS3	73.33	2		15	ZNF703	0.003561	
16	KLF10	74			16	STAT2	0.003686	4
17	CEBPD	75.6	5		17	ZNF205	0.003734	
18	ORCS8MEF2	76			18	IRX3	0.004274	2
19	CREB3L2	77.67	2		19	ZNF524	0.0043	
20	HMGN3	81.25	2		20	ETV6	0.004356	
21	PRRX2	91.67	3		21	AEBP1	0.004914	2
22	FOSL2	99.4	3		22	HMGN3	0.004978	
23	ZNF32	100.3			23	FOXQ1	0.004986	
24	EPAS1	100.5			24	ZNF32	0.0056	
25	SOX4	104			25	SOX2	0.006098	5
26	SNAPC5	107			26	GLIS3	0.006223	2
27	ATF6B	107	2		27	IRF1	0.006734	4
28	CREB3L1	107.3	3		28	BHLHE40	0.006757	5
29	IRF7	107.7	4		29	PRDM2	0.006845	
30	ZNF469	108	3		30	ATF5	0.007123	
31	GTF2B	109.8			31	PRRX2	0.007371	3
32	ZNF358	115.7			32	TSHZ2	0.007467	
33	ZNF883	116.5			33	SOX9	0.007835	
34	SOX9	116.6			34	GLIS1	0.007985	3
35	OSR2	117			35	SNAI1	0.00809	2
36	ZBED3	120.3			36	JUN	0.008475	4
37	ESR1	120.5			37	CREB3L2	0.0086	
38	ZNF503	122			38	NCOA1	0.008712	
39	ATF3	122.5	5		39	OSR2	0.009214	
40	KLF9	124.5			40	ST18	0.009334	
41	PGR	127.8			41	ASCL2	0.009828	
42	ZNF524	130.3	2		42	DDIT3	0.009956	5
43	STAT6	132.3			43	CEBPB	0.009972	5
44	TSHZ1	134.3			44	RELA	0.0101	2
45	HHEX	135.5			45	ZBTB14	0.01058	
46	IRF2	138.3	2		46	RELB	0.01068	3
47	RELB	142.5	5		47	NR1H3	0.01106	4
48	OSR1	143	2		48	SP100	0.0112	3
49	SNAI2	148.5	2		49	KLF11	0.01182	
50	BATF3	149	3		50	RFX5	0.01211	
335	ATF5				30	ATF5		
51	CEBPB				43	CEBPB		
17	CEBPD				60	CEBPD		

MDA-MB-231

	MEAN RANK						TOP RANK	
Rank	TF	Score	# Lines		Rank	TF	Score	# Lines
1	GLMP	4	5		1	ATF3	6.14E-04	3
2	CEBPB	15	5		2	RUNX1	6.22E-04	2
3	CSRNP1	17	3		3	HBP1	7.12E-04	2
4	ATF3	19	5		4	CSRNP1	0.001229	3
5	NFIL3	26	4		5	NR4A1	0.001245	
6	MZF1	27.33			6	DDIT3	0.001425	5
7	FOSB	27.33	3		7	FOSL2	0.001843	2
8	ARID5A	28.5	4		8	GLMP	0.001867	5
9	BHLHE40	31.25	6		9	MXD1	0.002137	3
10	JUN	34.5	4		10	JUNB	0.002457	5
11	RBCK1	35.5	3		11	ANKZF1	0.002849	3
12	HSF4	36			12	ETV4	0.003367	
13	ZNF76	41			13	BCL6	0.003561	2
14	PLSCR1	43	6		14	BHLHE40	0.003686	5
15	FOSL2	43.4	3		15	RAG1	0.003734	2
16	MBD6	46.5			16	JUND	0.0043	
17	ZNF395	48.67	4		17	EGR1	0.004356	2
18	JUNB	48.75	3		18	NR1H3	0.004914	4
19	CEBPD	49.8	5		19	ZNF676	0.004978	
20	SREBF1	50.5	2		20	MAFF	0.004986	2
21	ZNF580	50.67			21	FOSB	0.005528	3
22	ANKZF1	51.67	2		22	CREBL2	0.0056	2
23	KLF2	60			23	ZNF425	0.005698	
24	EGR1	61.83			24	SOX2	0.006098	5
25	SCX	62	2		25	ETS2	0.006143	3
26	NR4A1	62.25	3		26	PBX2	0.006223	2
27	IRF1	64.67	6		27	NFE2L2	0.006757	3
28	SNAI1	68.33	3		28	MZF1	0.006845	
29	RARA	76.4			29	CEBPB	0.007123	5
30	IRF9	76.75	5		30	MSC	0.007371	3
31	RELB	78.25	5		31	CREB3L1	0.007467	3
32	ETS2	78.5	2		32	NFIL3	0.007835	3
33	STAT3	80.83	5		33	CEBPD	0.007985	4
34	SAFB2	81.5			34	ZNF75D	0.00809	2
35	ZNF524	83.67	2		35	STAT3	0.008475	3
36	HLX	84	3		36	ZNF467	0.008547	3
37	PPARD	86.25	2		37	RXRA	0.0086	
38	CREBL2	86.33	2		38	ARID5A	0.009214	2
39	JDP2	97	3		39	MXI1	0.009259	2
40	IRF3	97.2			40	TSHZ2	0.009334	
41	TFE3	97.33			41	MXD4	0.009972	
42	FLYWCH1	98			42	MAZ	0.0101	2
43	ZBTB22	99			43	TBX3	0.01058	2
44	DDIT3	99	2		44	ZBTB22	0.01068	
45	MNT	99.5			45	JUN	0.01106	4
46	NFKB2	100.5	4		46	SOX4	0.0112	2
47	RXRB	101			47	ZNF554	0.0114	
48	BCL6	101.2	2		48	PPARD	0.01167	
49	ATF6B	101.5	2		49	SNAI1	0.01182	2
50	CREB3L1	102.3	3		50	ZNF385A	0.01211	2
364	ATF5				89	ATF5		
2	CEBPB				29	CEBPB		
19	CEBPD				33	CEBPD		

T98G

	MEAN RANK						TOP RANK	
Rank	TF	Score	# Lines		Rank	TF	Score	# Lines
1	PLSCR1	3.5	6		1	PLSCR1	6.14E-04	3
2	BATF2	3.667	4		2	IRF9	6.22E-04	3
3	IRF7	5.667	4		3	ETV7	0.001229	4
4	SP100	7.333	3		4	SP100	0.001245	3
5	IRF9	8	5		5	STAT2	0.001425	4
6	IRF1	12.33	6		6	STAT1	0.001843	4
7	STAT2	12.4	4		7	BATF2	0.001867	4
8	ZNF267	16.33	3		8	BATF3	0.002489	2
9	BATF3	21.67	3		9	IRF7	0.003111	3
10	ARID5A	25.5	4		10	TRAFD1	0.003686	3
11	CSRNP1	27.5	3		11	RELB	0.004356	3
12	STAT1	27.67	2		12	NFKB1	0.004914	3
13	CEBPB	28.67	5		13	NFKB2	0.004978	2
14	GLMP	33.5	5		14	MSC	0.005528	3
15	MSC	34.33	2		15	IRF1	0.0056	4
16	RELB	38	5		16	SP110	0.005698	2
17	STAT3	40.5	5		17	SOX2	0.006098	5
18	ATF3	40.83	5		18	ATF3	0.006143	3
19	JUNB	42.25	3		19	ZNF267	0.006223	2
20	SP140	44.33	2		20	SP140L	0.006845	2
21	TWIST2	46.5	3		21	NFE2L2	0.007371	3
22	EGR2	48	2		22	ARID5A	0.007467	2
23	BATF	48.8			23	HES4	0.007835	2
24	JUN	49.83	5		24	L3MBTL3	0.00809	
25	IRF8	50.25	2		25	MTF1	0.009214	
26	JDP2	51.33	3		26	MXD1	0.009259	3
27	AEBP1	51.5	2		27	TFEC	0.009334	2
28	IRF2	53.5	2		28	HLX	0.009956	
29	HLX	54.67	3		29	HESX1	0.009972	
30	TRAFD1	55	2		30	CEBPD	0.01058	4
31	SP140L	55	2		31	HDX	0.01068	
32	SP110	57.67	2		32	FOSB	0.0112	3
33	CEBPD	57.8	5		33	NFE2L3	0.0114	2
34	TFEC	59.67	2		34	BATF	0.01167	2
35	NFIL3	65	4		35	EGR2	0.01182	
36	FOSB	65.33	3		36	DDIT3	0.01211	5
37	FOS	67.4	2		37	RELA	0.0122	2
38	ZNF469	68	3		38	GLMP	0.01245	5
39	SNAI1	68.67	3		39	IRF2	0.01282	2
40	AHR	69.6	2		40	ETV3	0.0129	
41	NFKB2	70.5	4		41	AHR	0.01307	2
42	NR4A1	76.5	3		42	FOS	0.01347	2
43	STAT5A	80.83	3		43	NR3C1	0.01351	
44	ELF4	82			44	SP140	0.01353	2
45	RUNX2	82.6	2		45	PBX2	0.01369	
46	NR1H3	85.2	3		46	CSRNP1	0.01413	3
47	BCL6	86	2		47	JUNB	0.01425	5
48	KLF6	86.5	3		48	FLI1	0.01431	2
49	BHLHE40	89.75	6		49	IRF8	0.01474	2
50	PRDM1	91.8	2		50	ZNF467	0.01496	3
212	ATF5				52	ATF5		
13	CEBPB				51	CEBPB		
33	CEBPD				30	CEBPD		

A375

	MEAN RANK						TOP RANK	
Rank	TF	Score	# Lines		Rank	TF	Score	# Lines
1	AEBP1	5.5	2		1	ATOH8	6.14E-04	
2	TWIST2	13	3		2	PRRX2	6.22E-04	3
3	NFATC4	17.33	2		3	ZNF395	7.12E-04	3
4	ZNF469	17.5	3		4	GLIS1	0.001229	3
5	FOXC2	22.67			5	PRDM16	0.001245	
6	PRRX2	23.67	3		6	CEBPB	0.001425	5
7	GLIS2	24.67			7	MKX	0.001843	
8	FOXS1	25.33			8	FOXL1	0.001867	2
9	RFX8	27.5	2		9	ANKZF1	0.002137	3
10	FOXD1	29			10	BHLHE40	0.002489	5
11	SCX	31	2		11	JUNB	0.002849	5
12	HIF1A	36.6	3		12	AEBP1	0.003071	2
13	TWIST1	38			13	ERG	0.003111	
14	OSR1	40	2		14	ZBTB7A	0.003367	
15	ZNF395	45	4		15	MAFF	0.003561	2
16	NFIL3	50	4		16	CREB3L1	0.003686	2
17	HEYL	50.67			17	NFIL3	0.004274	3
18	HES4	52.33			18	PRRX1	0.0043	
19	SNAI2	53.25	2		19	FOXS1	0.004356	
20	PRRX1	57.67	2		20	TWIST2	0.004914	
21	HIC1	59.33			21	ELK3	0.004978	2
22	ATF3	64.33	5		22	TEAD2	0.004986	
23	FOXL1	67.67			23	ZNF469	0.005528	
24	SNAI1	68	3		24	TWIST1	0.0056	
25	BNC2	71.67			25	NFATC4	0.005698	
26	BHLHE40	79.5	6		26	HIF1A	0.006098	3
27	MSC	81	2		27	GLIS2	0.006143	
28	TBX18	84			28	FOXC2	0.006223	2
29	TEAD3	84.33			29	DDIT3	0.00641	5
30	CEBPB	85.67	5		30	STAT3	0.006734	3
31	MEIS3	86			31	ZNF358	0.007123	
32	IRF1	96.33	6		32	FOXF1	0.007371	2
33	SOX10	96.5			33	MSC	0.007467	3
34	MEOX1	97.33			34	ETS2	0.007835	3
35	NACC2	99			35	TBX18	0.007985	
36	MEOX2	100.3			36	HIC1	0.00809	
37	ELK3	107.5			37	GATA2	0.008475	
38	MKX	110			38	EGR1	0.008547	2
39	EGR2	115	2		39	NKX26	0.0086	
40	JDP2	118	3		40	ZNF385D	0.009334	
41	PLSCR1	118.5	6		41	SNAI2	0.009828	2
42	ZNF428	119			42	TCF7L1	0.009956	
43	PPARG	119.2	3		43	MAZ	0.0101	2
44	TCF21	119.3	2		44	FOXC1	0.01058	
45	NR2F2	119.4			45	JUN	0.01068	4
46	HLX	121.3	3		46	OSR1	0.01106	
47	NFE2L2	125.6	3		47	FOSB	0.0114	3
48	ZNF697	126.3	3		48	BNC2	0.01167	
49	JUN	127	5		49	HEYL	0.01182	
50	TEAD2	129			50	IRX3	0.01211	2
283	ATF5				66	ATF5		
30	CEBPB				6	CEBPB		
76	CEBPD				86	CEBPD		

A549

	MEAN RANK							TOP RANK	
Rank	TF	Score	# Lines	DEG LOG2FC		Rank	TF	Score	# Lines
1	PLSCR1	3.5	6	0.51		1	NFE2L2	6.14E-04	3
2	ZNF395	12.33	4			2	SOX4	6.22E-04	2
3	GLMP	13.5	5			3	ANKZF1	7.12E-04	3
4	NFIL3	23.33	4	0.87		4	CSRNP1	0.001229	3
5	CEBPB	29.17	5	0.82		5	TBX3	0.001245	2
6	STAT2	34.6	4	0.72		6	HBP1	0.001425	2
7	CEBPD	42.2	5			7	PLSCR1	0.001843	3
8	ARID5A	43.5	4	-2.58		8	CREBL2	0.001867	2
9	BHLHE40	45.5	6	0.95		9	ZNF277	0.002137	
10	STAT3	50.67	5			10	FOSL2	0.002457	2
11	FOSB	57.67	3			11	MXD1	0.002849	3
12	ATF3	60.33	5			12	CEBPD	0.003071	4
13	IRF1	60.5	6			13	SREBF1	0.003111	
14	JUNB	62	3	1.20		14	CEBPB	0.003367	5
15	CSRNP1	62.5	3			15	ZNF395	0.003561	3
16	ELF3	63.5	2			16	ETS2	0.003686	3
17	CREBL2	64.67	2	0.41		17	GLMP	0.003734	5
18	ETS2	64.75	2			18	BACH1	0.004274	
19	IRF9	64.75	5	0.54		19	BATF2	0.0043	4
20	JUN	65.83	5	-0.44		20	XBP1	0.004356	
21	NR4A1	68.75	3			21	NR1H3	0.004914	4
22	SP100	71	3	0.35		22	BCL6	0.004986	2
23	BATF2	75.33	4			23	BHLHE40	0.005528	5
24	RELB	77	5			24	RUNX1	0.0056	2
25	HIF1A	79.6	3	1.41		25	MXI1	0.005698	2
26	RBCK1	81.5	3			26	SOX2	0.006098	5
27	NR1H3	81.6	3	0.71		27	ATF3	0.006143	3
28	ZBED6	82				28	RAG1	0.006223	2
29	NKX21	85				29	ZNF467	0.00641	3
30	FOSL2	86.8	3			30	STAT1	0.006734	4
31	FOXO3	88.75				31	AHR	0.006757	2
32	AHR	94.8	2	0.63		32	ZNF280A	0.006845	
33	ANKZF1	99	2	0.78		33	JUNB	0.007123	5
34	BACH1	99		0.95		34	HIF1A	0.007371	3
35	FOS	101	2			35	CREB3L1	0.007467	3
36	FOXA1	102.5				36	IRF1	0.007835	4
37	CREB3L2	104.3	2			37	NFKB1	0.007985	3
38	SREBF1	106.5	2	1.02		38	ZNF75D	0.00809	2
39	NFE2L2	107.8	3			39	STAT3	0.008475	3
40	PPARG	113.4	3			40	GLIS3	0.008712	2
41	CEBPA	115				41	STAT2	0.009214	4
42	XBP1	117.3	2	1.25		42	NFIL3	0.009259	3
43	GLIS3	117.7	2			43	ZNF275	0.009334	
44	ZNF33B	117.7				44	KLF10	0.009828	
45	IRF7	118.7	4	1.06		45	ZNF69	0.009956	
46	PPARD	119.8	2			46	DDIT3	0.009972	5
47	ZNF267	123	3			47	FOXA2	0.0101	
48	TRPS1	123.3				48	ASCL3	0.01058	2
49	NFKB2	124.5	4			49	IRF2	0.01068	2
50	STAT5A	125.3	3			50	ETV7	0.01106	4
89	ATF5					340	ATF5		
5	CEBPB					14	CEBPB		
7	CEBPD					12	CEBPD		

MCF7

	MEAN RANK						TOP RANK	
Rank	TF	Score	# Lines		Rank	TF	Score	# Lines
1	IRF7	4.333	4		1	STAT1	6.14E-04	4
2	BATF2	10.67	4		2	IRF9	6.22E-04	3
3	STAT2	11.6	4		3	IRF7	7.12E-04	3
4	PLSCR1	13	6		4	PLSCR1	0.001229	3
5	SP100	16	5		5	NFKB2	0.001245	2
6	IRF9	23.75	5		6	ETV7	0.001843	4
7	IRF1	28	6		7	BATF3	0.001867	2
8	SP140	29	2		8	STAT2	0.002457	4
9	ELF3	29.5	2		9	TFEC	0.002489	2
10	STAT1	29.67	2		10	SP140L	0.003111	2
11	BHLHE40	31.25	6		11	FOSL1	0.003686	
12	BATF3	32.33	3		12	SP100	0.003734	3
13	TWIST2	37.5	3		13	IRF1	0.004274	4
14	HIF1A	42.6	3		14	TRAFD1	0.0043	3
15	ZNF395	43.67	4		15	BATF	0.004914	2
16	PRDM1	51.6	•		16	IRF8	0.004978	2
17	NFKB2	56.75	4		17	BATF2	0.004986	4
18	IRF8	58.25	2		18	SNAI2	0.005528	2
19	ZNF267	59.67	3		19	PRDM1	0.0056	
20	PPARG	70.2	3		20	ZNF395	0.005698	3
21	RBCK1	74	3		21	HIF1A	0.006098	3
22	PRRX2	76.67	3		22	BHLHE40	0.006223	5
23	ATF5	80.67	2		23	SP110	0.00641	2
24	SP140L	81	2		24	ELK3	0.006845	2
25	HMGN3	81.75	2		25	CEBPB	0.007123	5
26	EHF	83.75			26	GLMP	0.007371	5
27	GLMP	84.5	5		27	PHOX2A	0.007467	
28	ARNTL2	86.67			28	FOXF1	0.007985	2
29	STAT3	87.5	5		29	ETS1	0.00809	
30	FOSL1	90.4			30	HES4	0.008547	2
31	RELB	93.25	5		31	PRRX2	0.0086	3
32	PRRX1	94	2		32	NR1H3	0.009259	4
33	RFX5	95.4			33	ZNF267	0.009334	2
34	CEBPB	96.33	5		34	FOXC2	0.009828	2
35	CREB3L1	97.25	3		35	ZNF562	0.009956	
36	RUNX2	100.8	2		36	NFE2L3	0.009972	2
37	NFATC4	102	2		37	FOXL1	0.01044	2
38	NFE2L2	102.2	3		38	SP140	0.01058	2
39	JUN	103.2	5		39	JUNB	0.01068	5
40	STAT5A	103.7	3		40	GLIS1	0.01106	3
41	TFEC	104.7	2		41	CEBPD	0.0112	4
42	NR1H3	110.6	3		42	RELB	0.0114	3
43	HOXB5	110.7			43	TCF21	0.01167	
44	CEBPD	113.2	5		44	DMRTA2	0.01182	
45	TCF21	113.8	2		45	FOS	0.01211	2
46	IRF5	114.7			46	SOX2	0.0122	5
47	RUNX3	116.2			47	FLI1	0.01245	2
48	ARID5A	120.5	4		48	TBX4	0.01307	
49	KLF6	120.5	3		49	EPAS1	0.01347	
50	RFX8	121	2		50	NFKB1	0.01351	3
23	ATF5				51	ATF5		
34	CEBPB				25	CEBPB		
44	CEBPD				41	CEBPD		

Figure S6. ChEA3 Transcription Factor Enrichment Analysis (TFEA) of upregulated DEGs induced by Dpep in six cancer cell lines. Transcription Factor Enrichment Analysis (TFEA) was carried out by ChEA3 on upregulated DEGs for each cell line. The top 50 ranking results from both the Top Rank and Mean Rank libraries are shown for each line. For each cell line, the rankings and scores are given for each TF, as well as the numbers of lines in which the specific TF is ranked in the same library. For each line, TFs that appear in both libraries are highlighted in yellow. Ranks and scores for ATF5, CEBPB and CEBPD are highlighted in green.
